# Women and TB/HIV coinfection in Brazil: regional inequalities and trends in a scenario of vulnerability

**DOI:** 10.1590/0034-7167-2025-0259

**Published:** 2026-02-23

**Authors:** Emillyn Vitoria de Oliveira, Jarbas da Silva Ziani, Laís Mara Caetano da Silva Corcini, Flávia Meneguetti Pieri, Josilene Dália Alves, Emiliana Cristina Melo, Ricardo Castanho Moreira, Alessandro Rolim Scholze

**Affiliations:** I Universidade Estadual do Norte do Paraná. Bandeirantes, Paraná, Brazil.; II Universidade Federal de Santa Maria. Santa Maria, Rio Grande do Sul, Brazil.; III Universidade Estadual de Londrina. Londrina, Paraná, Brazil.; IV Universidade Federal do Mato Grosso. Cuiabá, Mato Grosso, Brazil.

**Keywords:** Women, Coinfection, Tuberculosis, HIV, Spatial Analysis

## Abstract

**Objectives:**

to identify the clinical-epidemiological profile of women with TB/HIV coinfection and classify the temporal trend of coinfection in the regions of Brazil.

**Methods:**

ecological study of time series of records from the Notifiable Diseases Information System carried out from 2012 to 2023. The analysis was performed using the Prais-Winsten autoregression method.

**Results:**

a total of 31,171 cases were recorded in the country, with the highest concentration in the Southeast. Coinfection showed a steady trend, with higher rates in the South and North regions. Regional disparities are related to factors such as low education level, age between 20 and 39 years, and black race/skin color. In the South, drug resistance and substance use disorders also stood out.

**Conclusions:**

the need for regional, equitable and integrated public policies is evident, focusing on expanding access to diagnosis and treatment, considering the specific vulnerabilities of women affected by coinfection.

## INTRODUCTION

Tuberculosis (TB) is an infectious disease of ancient origin, preventable and curable. However, it is estimated that approximately one-third of the world's population is infected with *Mycobacterium tuberculosis*. Furthermore, in 2023, TB accounted for 7.5 million deaths worldwide, surpassing deaths from COVID-19, becoming the leading cause of death from infectious diseases on the planet^1^. In addition, the Human Immunodeficiency Virus (HIV) epidemic, which emerged in the 1980s, significantly impacted the epidemiological profile and control of TB, making this TB/HIV co-infection one of the greatest challenges for public health^2^.

TB/HIV co-infection is even more concerning because the interaction between these diseases potentiates their harmful effects by compromising the function of immune T cells and macrophages. This reduces the levels and activity of lymphocytes (LT CD4+), promoting the progression of Acquired Immunodeficiency Syndrome (AIDS) due to immunosuppression and the emergence of opportunistic infections, TB being the most serious of these^3^.

In Brazil, between 2001 and 2020, 133,830 new cases of TB-HIV coinfection were identified. In 2023, 1,724 deaths attributed to coinfection were reported, which corresponds to an average of approximately five deaths per day^4^.

When analyzing the gender disparity in TB/HIV coinfection, a review study identified that women, especially younger women, bear a disproportionately greater burden compared to men as well as being more prone to death^5^. Furthermore, TB/ HIV co-infection is strongly associated with precarious living, health and work conditions, mainly affecting populations in situations of social vulnerability, including homeless people, people deprived of liberty, people with low levels of education and women^3^.

Therefore, identifying the sociodemographic profile of women affected by this coinfection is essential for directing more effective control policies, focusing on expanding access to early diagnosis, timely treatment, and prevention strategies. Furthermore, analyzing these profiles allows for directing efforts to areas of greater vulnerability and improving the operationalization of TB/HIV control programs, ensuring a more equitable and efficient response to the disease^6^.

Thus, the study is justified given the relevance and lack of studies that address TB/HIV co-infection among women at a national level as well as that apply the Prais-Winsten technique^7^. It is known that TB/HIV coinfection presents distinct epidemiological profiles across Brazilian regions. For instance, while the North region historically records high incidence of TB, the South and Southeast regions may present specific challenges related to population density and overloaded healthcare services, reinforcing the need for in-depth analyses on a national scale^3^. The magnitude of TB as the leading cause of death from infectious disease worldwide, combined with the impacts of HIV on the immune response, creates a critical scenario for controlling co- -infection, which disproportionately affects women, especially younger women in socially vulnerable situations. Furthermore, developing countries like Brazil deserve special attention from this perspective, as social inequality represents fertile ground for the spread of co-infection, especially among women.

## OBJECTIVES

To identify the clinical-epidemiological profile of women with TB/HIV coinfection and classify the temporal trend of coinfection in the regions of Brazil.

## METHODS

### Ethical aspects

This study does not require prior approval from the Research Ethics Committee, nor does it require an Informed Consent Form, since it uses freely accessible secondary data available in the Brazilian Health System information systems. However, we reiterate our commitment to Resolutions 466/12 and 510/16 regarding data collection and analysis to maintain participant anonymity.

### Study design

This is an ecological time series study, guided by the STrengthening the Reporting of OBservational studies in Epidemiology tool.

### Study site

The study was conducted across Brazil’s entire territory (8,515,767,049 km2), according to the Brazilian Institute of Geography and Statistics, with an estimated population of 215.6 million inhabitants in 2023. Brazil is divided into 26 states and one Federal District, which together form the 27 federative units. These, in turn, are arranged into five geographic regions: North, Northeast, South, Southeast, and Midwest.

### Study period

For the study, data available from January 1, 2012 to December 31, 2023 were considered, while data collection and analysis was conducted from August to November 2024 and updated in March 2025.

### Data collection

Secondary data were obtained from the Notifiable Diseases Information System (In Portuguese, *Sistema de Informação de Agravos de Notificação* - SINAN) website, through DATASUS, of the Health Surveillance Department of the Ministry of Health (https://datasus.saude.gov.br/informacoes-de-saude-tabnet/).

### Population definition

Women diagnosed with TB/HIV coinfection, with the AIDS variable coded as “yes” in the TB/HIV coinfection reports registered in SINAN, and residing in regions belonging to Brazilian states at the time of diagnosis were included. Ignored cases and/or those with blank data were excluded.

### Study variables

The dependent variable for our time-series analysis was the annual number of TB/HIV coinfection cases in women, stratified by each of the five major regions of Brazil (North, Northeast, Southeast, South, and Midwest). In SINAN, TB/HIV coinfection is defined as TB diagnosis in individuals with concomitant HIV infection.

As independent variables, we considered the year of report (for time trend analysis) and a set of characterization variables, which we divided into two groups. The first group contained sociodemographic characteristics and comorbidities, such as age group (<1 year, 1 to 19, 20 to 39, 40 to 59, 60 years or older), race/ color (white, black, yellow, indigenous), education (illiterate, up to eight years of education, or eight years or more), alcohol use disorder (yes/no), illicit substance use disorder (yes/no), mental disorder (yes/no) and tobacco use disorder (yes/no). The second group contained the following clinical characteristics: entry type is characterized by the way the case was entered into the report system (new case, recurrence, transfer, post-mortem); clinical form includes TB anatomical location classification (pulmonary, extrapulmonary, or pulmonary and extrapulmonary); laboratory confirmation refers to the presence (with confirmation) or absence (without confirmation) of a laboratory result that confirms the diagnosis; antiretroviral treatment (ART) includes the record of the use of antiretroviral therapy for people living with HIV (yes/ no); closure status encompasses the final outcome of the TB case in the system (cure, treatment abandonment, death from TB, death from other causes, transfer, drug-resistant tuberculosis (DR-TB), or other outcome).

### Data analysis

Descriptive analyses of absolute and relative frequencies regarding sociodemographic characteristics, comorbidities, and clinical profile were performed using the IBM Statistical Package for the Social Sciences version 20.

To analyze the temporal trend of TB/HIV coinfection in women across different regions of Brazil, we used the Prais-Winsten regression method^7^. This model is widely used in time series analyses to correct for autocorrelation of residuals, a common feature in sequential epidemiological data, thus ensuring the validity of estimates^7^. In the model, the annual number of TB/HIV coinfection cases in women in each region was the dependent variable, and the year of report was the independent variable.

Temporal trend was expressed as Annual Percentage Change (APC), with 95% Confidence Intervals (95%CI). The trend was interpreted based on a 5% significance level (p < 0.05) and a 95%CI of APC. A stationary trend was considered when APC 95%CI contained zero and p-value was greater than 0.05, indicating no significant variation over time. An increasing trend was classified when APC 95%CI did not contain zero, the APC value was positive, and p < 0.05. A decreasing trend was classified when the APC 95%CI did not contain zero, the APC value was negative, and p < 0.05. All time series analyses were performed in Stata version 14.

## RESULTS

Between 2012 and 2023, 31,171 cases of TB/HIV co-infection were reported among women nationwide, with the Southeast region having the highest number of reports, with 11,791 cases (37.8%), followed by the South region, with 7,958 cases (25.5%), Northeast, with 6,788 cases (21.7%), North, with 3,340 cases (10.7%), and Midwest, with 1,294 cases (4.1%).

When analyzing the sociodemographic characteristics and comorbidities associated with the reporting regions, the age group of 20 to 39 years and up to eight years of education were the most prevalent variables in the five regions of Brazil. Black people were more frequently reported in the North, Northeast, Southeast, and Midwest regions, and white people in the South. Regarding comorbidities, in the Midwest region, alcohol use disorder was most prevalent (24.5%), followed by tobacco use disorder (29.8%). In the South region, illicit substance use disorder (36.7%) and tobacco use disorder (39.5%) were the most prevalent, as described in Table 1.

**Table 1:** Characterization of the sociodemographic profile and comorbidities associated with TB/HIV coinfection in women according to the regions of Brazil, 2012-2023

Variables	North n(%)	Northeast n(%)	Southeast n(%)	South n(%)	Midwest n(%)
**Age range (years)** *					
<1 year	7(0.2)	47(0.7)	31(0.3)	12(0.2)	4(0.3)
1-19	140(4.2)	235(3.5)	376(3.2)	156(2.0)	23(1.8)
20-39	1784(53.4)	3677(54.2)	5890(50.0)	4273(53.7)	634(49.0)
40-59	1257(37.6)	2550(37.6)	4905(41.6)	3163(39.7)	546(42.2)
>60	152(4.6)	279(4.1)	589(5.0)	354(4.4)	87(6.7)
**Education (years of study)** *					
Illiterate	121(4.7)	615(13.6)	718(8.6)	403(6.3)	78(8.5)
<8	1507(58.0)	2918(64.5)	4622(55.1)	4538(71.5)	558(60.5)
>8	1069(41.2)	989(21.9)	3042(36.3)	1410(22.2)	286(31.0)
**Race/color** *					
White	240(7.4)	639(10.2)	3429(32.1)	4650(59.8)	270(21.8)
Black	2947(91.2)	5559(89.0)	7165(67.0)	3089(39.7)	935(75.6)
Yellow	20(0.6)	36(0.6)	74(0.7)	34(0.4)	7(0.6)
Indigenous	23(0.7)	11(0.2)	26(0.2)	8(0.1)	25(2.0)
**Alcohol use disorder** *					
Yes	504(16.0)	1412(23.7)	2026(18.7)	1625(21.2)	276(24.5)
No	2641(84.0)	4546(76.3)	8807(81.3)	6042(78.8)	849(75.5)
**Substance use disorder** *					
Yes	441(17.6)	1236(26.7)	2607(27.1)	2198(36.7)	228(25.1)
No	2067(82.4)	3398(73.3)	7030(72.9)	3788(63.3)	679(74.9)
**Tobacco use disorder** *					
Yes	413(16.4)	1092(23.9)	2086(21.7)	2352(39.5)	270(29.8)
No	2099(83.6)	3476(76.1)	7506(78.3)	3595(60.5)	635(70.2)

* *Ignored and blank variables were excluded from the analysis*.

Table 2 shows the clinical profile of TB/HIV coinfection. Thus, new cases, pulmonary clinical forms, and those on ART were more prevalent in the five regions of Brazil. Regarding laboratory confirmation of TB, only the Southeast and South regions presented laboratory confirmation. Concerning the resolution of cases, cure was evidenced in a higher proportion in all five regions. However, treatment abandonment and DR-TB were more prevalent in the South region (27.9% and 2.0%, respectively), death from TB in the Southeast region (4.9%), and deaths from other causes in the Midwest region (21.1%).

**Table 2:** Clinical profile of TB/HIV coinfection in women according to regions of Brazil, 2012-2023

Variables	North n(%)	Northeast n(%)	Southeast n(%)	South n(%)	Midwest n(%)
**Entry type** *					
New case	2478(74.3)	4346(64.5)	7972(68.1)	4809(60.7)	951(74.3)
Recurrence	728(21.8)	1936(28.7)	3260(27.8)	2683(33.8)	262(20.5)
Transfer	114(3.4)	391(5.8)	319(2.7)	386(4.9)	58(4.5)
Post-mortem	15(0.4)	63(0.9)	157(1.3)	50(0.6)	9(0.7)
**Clinical form** *					
Pulmonary	2328(69.7)	4932(72.7)	8327(70.6)	5444(68.4)	847(65.5)
Extrapulmonary	569(17.0)	1318(19.4)	2262(19.2)	1601(20.1)	311(24.0)
Pulmonary + extrapulmonary	443(13.3)	538(7.9)	1201(10.2)	913(11.5)	136(10.5)
**Laboratory confirmation** *					
With confirmation	1545(46.3)	3004(44.3)	6221(52.8)	4460(56.0)	537(41.5)
Without confirmation	1795(53.7)	3784(55.7)	5570(47.2)	3498(44.0)	757(58.5)
**Antiretroviral treatment** *					
Yes	1582(80.1)	2592(72.5)	4471(74.5)	3431(66.1)	667(81.5)
No	392(19.9)	984(27.5)	1529(25.5)	1759(33.9)	151(18.5)
**Closing status** *					
Cure	1483(46.5)	2293(35.2)	5249(46.0)	3077(39.3)	440(35.9)
Abandonment	677(21.2)	1673(25.7)	2570(22.5)	2189(27.9)	270(22.0)
Death from tuberculosis	86(2.7)	303(4.7)	555(4.9)	99(1.3)	43(3.5)
Death from other causes	578(18.1)	980(15.0)	2091(18.3)	1352(17.2)	258(21.1)
DR-TB**	47(1.5)	58(0.9)	164(1.4)	158(2.0)	12(1.0)
Other outcome	321(10.1)	1207(18.5)	779(6.8)	964(12.3)	202(16.5)

* *Ignored and blank variables were excluded from the analysis; DR-TB - drug-resistant tuberculosis*.

**Figure 1:**
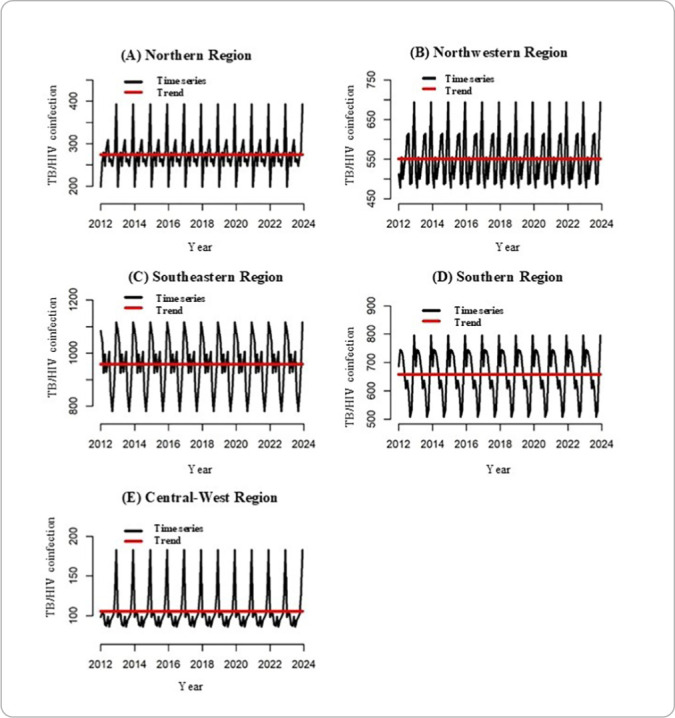
Time series decomposition of TB/HIV coinfection in Brazil: stationary increasing trend in all regions, 2012-2023

The time series decomposition technique (Figure 1) showed an increase in the temporal trend of stationary TB/HIV coinfection for all regions of Brazil. These findings support the data presented in Table 3 regarding the Prais-Winsten analysis results.

**Table 3:** Temporal trends of TB/HIV coinfection in women according to regions of Brazil, 2012-2023

Region	Reports	Rate	APC (95%CI)	*p* value	Trend
North	3296	37.92	26.38 (-1.20 - 80.27)	0.172	Stationary
Northeast	6611	23.41	1.03 (-1.55 - 3.68)	0.398	Stationary
Southeast	11494	26.13	-0.09 (-0.22 - 0.02)	0.096	Stationary
South	7899	51.45	-0.07 (-0.20 - 0.05)	0.209	Stationary
Midwest	1267	15.29	5.83 (-2.64 - 15.04)	0.161	Stationary

*APC - Annual Percentage Change; 95%CI - 95% Confidence Interval*.

Furthermore, through Prais-Winsten autoregression, from 2012 to 2023, it was possible to observe that TB/HIV coinfection remained with a stationary trend, observing a higher coinfection rate in the South region, followed by the North, as described in Table 3.

## DISCUSSION

This study aimed to identify the operational clinical profile and classify the temporal trend among women with TB/HIV coinfection in the regions of Brazil, in order to support prevention and control strategies aimed at the actions of public policy-making bodies,.

Analysis of the 31,171 reported cases of TB/HIV coinfection among women in Brazil revealed a significant concentration in the Southeast region, responsible for 37.8% of the cases, followed by the South (25.5%) and Northeast (21.7%) regions. However, this rate may also reflect the region’s higher population density and a more consolidated health report and surveillance structure.

By analyzing the unequal distribution of cases found, it becomes possible to highlight not only the regional disparities in the detection of TB/HIV coinfection, but also the structural nature of the social vulnerability faced by these Brazilian women^(8)^. Furthermore, the TB/HIV co-infection scenario is intrinsically linked to conditions of poverty, low education, lack of support network and exclusion from the health system, which increases morbidity and mortality among women, especially young women^(9,10)^.

In this context, it is important to consider that the higher proportion of TB/HIV coinfection reports among women can be explained by aspects of their sociohistorical background, marked by a greater appreciation for self-care and a more frequent search for healthcare services compared to men. This behavior favors early diagnosis and timely initiation of treatment. Studies conducted in South Africa and Uganda support this difference in outcomes between the sexes, indicating that men are more likely to die from TB than women^(11,12)^. Thus, identifying the clinical-operational profile of these women becomes essential for redirecting public policies and formulating more equitable strategies that are sensitive to local and gender specificities.

Regarding the age range of the study participants, which included the Southeast region, a concentration was observed between 20 and 39 years old. Corroborating this finding, a study conducted in the state of Rio de Janeiro, Brazil, between 2000 and 2016, also identified a higher proportion of cases in this same age group^(13)^. At an international level, a study in sub-Saharan Africa, in turn, recorded a higher prevalence in the 30 to 49 age group in women with TB/HIV coinfection^(14)^.

This concentration in younger women may be related to the period of heightened hormonal activity, which is associated with increased sexual desire and increased frequency of sexual intercourse. These characteristics increase exposure to risk factors such as multiple partners, difficulty negotiating condom use, sexual violence, and substance use, all of which contribute to HIV infection^(15)^.

Concerning education, the highest proportion of reports occurred among women with up to eight years of education. This group tends to have less access to essential information about TB and HIV prevention, which encourages risky behaviors, such as unprotected sex and difficulty recognizing early symptoms of infection^(6)^. Furthermore, lower levels of education are associated with greater exposure to vulnerability factors, including unprotected sex, which increases the risk not only of unwanted pregnancy, but also of sexually transmitted infections, including HIV^(16)^.

In India, it was found that women belonging to communities with higher levels of wealth, education and employment had greater knowledge about the HIV virus, which was associated with lower infection rates^(17)^. These findings, which reflect the realities of socioeconomic inequality and cultural barriers present in large and diverse countries like Brazil, reinforce that such barriers significantly limit access to healthcare services, contributing to late diagnoses and lower treatment adherence. Furthermore, low educational attainment is often associated with precarious living conditions, which favor the spread of TB, as well as with social stigma that hinders the pursuit of appropriate care^(17)^.

In the Brazilian context, similar patterns are observed, where the highest rates of coinfection often fall on more socioeconomically vulnerable populations, including black women, despite the white population being larger, which requires understanding the contextual differences in dealing with the disease^(18)^. These vulnerabilities are reflected in the regions with the highest reports and indicate a temporal trend linked to social exclusion. Stigma and structural barriers hinder the search for care and increase morbidity and mortality. Therefore, coping strategies must consider these inequalities to improve the response to coinfection^(19)^.

In Brazil, the non-white population was less affected by TB/ HIV coinfection, a pattern also observed in countries such as the United States and Puerto Rico^(20)^. This greater susceptibility is associated with contexts of socioeconomic vulnerability, in which black populations face structural barriers, such as limited access to healthcare and education services, in addition to persistent racial discrimination(21). Furthermore, studies indicate that, in Brazil, the likelihood of experiencing sexual violence in adulthood is significantly higher among black women compared to other groups. This situation not only exposes them to a greater risk of HIV infection but can also create significant barriers to access and adherence to treatment and prevention of TB/HIV co-infection, negatively impacting health outcomes^(22)^.

Despite national and international guidelines for TB control, the persistence of new cases highlights significant limitations in surveillance and monitoring systems, as well as social determinants that hinder access to timely diagnosis and treatment^(23)^. These aspects were highlighted by the results of the present study, which point not only to the continuity of the transmission chain, but also to the complexity involved in eradicating the disease in contexts of vulnerability.

The extrapulmonary form of TB is more prevalent among people living with HIV, since immunosuppression favors the spread of the bacillus beyond the lungs^(24)^. Moreover, 27% of women with TB/HIV infection presented this clinical form, reinforcing the need for specialized care and sensitive diagnostic strategies for early identification of these cases. This scenario requires not only strengthening epidemiological surveillance efforts but also expanding healthcare service coverage and training teams for the clinical and operational management of coinfection, especially in women in socially vulnerable situations.

Despite remarkable advances in combined treatment for TB/ HIV coinfection, including effective TB regimens and the widespread availability of ART, essential for achieving a cure, treatment discontinuation remains a critical challenge. Even with progress observed in regions such as the Midwest region, discontinuation is often linked to personal factors, such as alcohol, tobacco, and illicit drug use disorders, and to structural flaws in healthcare services, such as limited access and low treatment coverage. This scenario favors the emergence of resistant forms of the disease.

Furthermore, HIV is a significant predictor of treatment discontinuation and mortality. Comorbidities such as diabetes, mental disorders, and substance use increase women’s vulnerability to unprotected sex and TB/HIV coinfection, requiring a more integrated and humanized approach to care^(25)^.

Regional disparities have a direct impact on the incidence and outcomes of TB/HIV coinfection in Brazil, being most evident in the North and Northeast regions, which have historically been less developed. Given the country’s continental size, these imbalances are exacerbated by factors such as limited access to healthcare services, a lack of qualified professionals, inadequate distribution of medicines and supplies, and the difficulties imposed by geographic and socioeconomic barriers, contributing to the persistence of inequities^(26)^.

The scarcity of ecological studies on the spatial distribution of TB/HIV coinfection specifically among women limits a thorough understanding of the problem at the national level. However, the data obtained in this study clearly demonstrate regional inequalities in Brazil, reinforcing the importance of directing more research on this topic. It is essential to emphasize that, despite the existence of robust HIV and TB care programs that offer targeted treatment and follow-up, challenges remain in overcoming these regional inequities and ensuring equitable access to care. It is important to consider that, for the youngest and oldest groups in the age groups studied, the specificities of care, including the need for a guardian, are crucial. Therefore, it is essential to consider subjective, geographic, and socioeconomic aspects to increase treatment adherence and strengthen public policies.

### Study limitations

Limitations of this study include the fact that it is a retrospective analysis based on secondary data from SINAN. Although SINAN is a valuable official source, the quality and completeness of the information depend on completion at the time of reporting, which can lead to incomplete or missing variables.

This intrinsic characteristic of the database, combined with the possibility of underreporting, may result in an underestimation of the true magnitude of the problem and, consequently, affect the generalizability of the findings. Furthermore, the inherent limitation of ecological studies in individual inferences must be considered, which requires caution in interpretation to avoid ecological fallacy. Finally, the delay in making more recent data available in the system may have impacted the timeliness of the information analyzed.

### Contributions to health, nursing or public policies

This study contributes to public health by highlighting the social, clinical, and regional determinants that influence TB/HIV coinfection among Brazilian women, guiding more equitable and regionalized policies. For nursing, the importance of its role in early diagnosis, treatment adherence, and ongoing monitoring is highlighted, especially in vulnerable contexts, since nurses play a key role in managing healthcare services and in the care plan for this population.

## CONCLUSIONS

TB/HIV coinfection among women in Brazil presents regional disparities and is strongly associated with sociodemographic factors such as low education level, age range 20-39, and black race/skin color, especially in the North, Northeast, Southeast, and Midwest regions. The Southeast region concentrated the highest number of reports, but the South region had the highest rates of discontinuation and drug resistance, as well as a higher prevalence of substance use disorders.

The predominant clinical profile was new cases, pulmonary form, and those on ART. The temporal trend of co-infection remained stable between 2012 and 2023, with a notable increase in the rate in the South and North regions. Therefore, the findings of this study provide valuable contributions to the development of public policies targeted at women, considering the particularities of TB/HIV co-infection in each Brazilian region.

## Data Availability

The research data are available within the article.
