# Supplementary material for: Women and TB/HIV coinfection in Brazil: regional inequalities and trends in a scenario of vulnerability
Source: Rev Bras Enferm. 2026 Feb 23;79:e20250259. doi: 10.1590/0034-7167-2025-0259 (PMC12933940; doi:10.1590/0034-7167-2025-0259)
Supplement: Translation

# Translation: Mulheres e coinfecção TB/HIV no Brasil: desigualdades regionais e tendências em um cenário de vulnerabilidade

**DOI:** 10.1590/0034-7167-2025-0259pt

**Authors:** Emillyn Vitoria de Oliveira, Jarbas da Silva Ziani, Laís Mara Caetano da Silva Corcini, Flávia Meneguetti Pieri, Josilene Dália Alves, Emiliana Cristina Melo, Ricardo Castanho Moreira, Alessandro Rolim Scholze

**Affiliations:** I Universidade Estadual do Norte do Paraná. Bandeirantes, Paraná, Brasil.; II Universidade Federal de Santa Maria. Santa Maria, Rio Grande do Sul, Brasil.; III Universidade Estadual de Londrina. Londrina, Paraná, Brasil.; IV Universidade Federal do Mato Grosso. Cuiabá, Mato Grosso, Brasil.

**Keywords:** Mulheres, Coinfecção, Tuberculose, HIV, Análise Espacial

## Abstract

**Objectives:**

identificar o perfil clínico-epidemiológico das mulheres com coinfecção TB/HIV e classificar a tendência temporal da coinfecção nas regiões do Brasil.

**Métodos:**

estudo ecológico de séries temporais de registros do Sistema de Informação de Agravos de Notificação realizado no período de 2012 a 2023. A análise foi realizada utilizando o método de autorregressão Prais-Winsten.

**Resultados:**

foram registrados 31.171 casos no país, com maior concentração no Sudeste. A coinfecção apresentou tendência estacionária, com taxasmais altas nas regiões Sul e Norte. As disparidades regionais se relacionam a fatores comobaixa escolaridade, idade entre 20 e 39 anos, e raça/cor preta. No Sul, destacaram-se ainda a resistência medicamentosa e transtornos por uso de substâncias.

**Conclusões:**

evidencia-se a necessidade de políticas públicas regionais, equitativas e integradas, com foco na ampliação do acesso ao diagnóstico e tratamento, considerando as vulnerabilidades específicas dasmulheres afetadas pela coinfecção.

## Introdução

A tuberculose (TB) é uma doença infecciosa de origem milenar previsível e curável. No entanto, estima-se que aproximadamente um terço da população mundial esteja infectada pelo *Mycobacterium tuberculosis* ^1^. Além disso, em 2023, a TB foi responsável por 7,5 milhões de mortes no mundo, superando os óbitos do da COVID-19, tornando-se a principal causa de morte por doenças infecciosas no planeta^1^. Somado a isso, a epidemia do Vírus da Imunodeficiência Humana (HIV), que surgiu na década de 1980, impactou significativamente o perfil epidemiológico e o controle da TB, tornando essa coinfecção TB/HIV um dos maiores desafios para a saúde pública^2^.

A coinfecção TB/HIV é ainda mais preocupante, pois a interação entre essas doenças potencializa seus efeitos nocivos ao comprometer a função das células T imunes e dos macrófagos. Isso reduz os níveis e a atividade dos linfócitos (LT CD4+), propiciando a progressão da Síndrome da Imunodeficiência Adquirida (AIDS), em razão da imunossupressão e do surgimento de infecções oportunistas, sendo a TB a mais grave delas^3^. No Brasil, entre 2001 e 2020, foram identificados 133.830 novos casos de coinfecção TB-HIV. Já em 2023, foram notificadas 1.724 mortes atribuídas à coinfecção, o que corresponde a uma média de aproximadamente cinco óbitos por dia^(4)^.

Ao analisar a disparidade de sexo na coinfecção TB/HIV, estudo de revisão identificou que as mulheres, principalmente as mais jovens, suportam uma carga desproporcionalmente maior em relação aos homens, bem como possuem maior propensão ao óbito^5^. Ademais, a coinfecção TB/HIV está fortemente associada a condições de vida, saúde e trabalho precárias, atingindo principalmente populações em situação de vulnerabilidade social, incluindo pessoas em situação de rua, privadas de liberdade, com baixa escolaridade e mulheres^(3)^.

Portanto, identificar o perfil sociodemográfico das mulheres acometidas por essa coinfecção é essencial para direcionar políticas de controle mais eficazes, focando na ampliação do acesso ao diagnóstico precoce, tratamento oportuno e estratégias de prevenção. Além disso, a análise desses perfis permite orientar esforços para áreas de maior vulnerabilidade e aprimorar a operacionalização dos programas de controle da TB/HIV, garantindo uma resposta mais equitativa e eficiente no enfrentamento da doença^(6)^.

Desse modo, o estudo justifica-se diante da relevância e da inexistência de estudos que abordem a coinfecção TB/HIV entre mulheres a nível nacional, bem como que aplique técnica de Prais-Winsten^7^. É sabido que a coinfecção TB/HIV apresenta perfis epidemiológicos distintos entre as regiões brasileiras. Por exemplo, enquanto a região Norte historicamente registra altas incidências de TB, as regiões Sul e Sudeste podem apresentar desafios específicos relacionados à densidade populacional e à sobrecarga de serviços de saúde, reforçando a necessidade de análises aprofundadas em escala nacional^3^. A magnitude da TB como principal causa de morte por doença infecciosa no mundo, somada aos impactos do HIV sobre a resposta imunológica, configura um cenário crítico para o controle da coinfecção que afeta, de maneira desproporcional, as mulheres, em especial as mulheres mais jovens em situação de vulnerabilidade social.

Ainda, nessa perspectiva, países em desenvolvimento, como o Brasil, merecem destaque, pois a desigualdade social representa um terreno fértil para disseminação da coinfecção, principalmente entre as mulheres.

## OBJETIVOS

Identificar o perfil clínico-epidemiológico das mulheres com coinfecção TB/HIV e classificar a tendência temporal da coinfecção nas regiões do Brasil.

## MÉTODOS

### Aspectos éticos

Este estudo dispensa a aprovação prévia do Comitê de Ética em Pesquisa para sua execução, bem como o Termo de Consentimento Livre e Esclarecido, visto que utiliza dados secundários de acesso livre disponíveis nos sistemas de informação do Sistema Único de Saúde. Todavia, reitera-se o compromisso com as Resoluções no 466/12 e no 510/16 no que tange à coleta e análise para manter o anonimato dos participantes.

### Delineamento do estudo

Estudo ecológico de séries temporais, guiado pela ferramenta *STrengthening the Reporting of OBservational studies in Epidemiology*.

### Local do estudo

O estudo foi realizado no território brasileiro em sua totalidade (8.515.767,049) km2, de acordo com o Instituto Brasileiro de Geografia e Estatística, com uma população estimada em 215,6 milhões de habitantes em 2023. O Brasil é distribuído em 26 estados e um Distrito Federal, que, juntos, formam as 27 unidades federativas. Essas, por sua vez, estão dispostas em cinco regiões geográficas: Norte, Nordeste, Sul, Sudeste e Centro-Oeste.

### Período

Para o estudo, foram considerados os dados disponíveis no período de 01 de janeiro de 2012 a 31 de dezembro de 2023, enquanto a coleta e análise de dados foram conduzidas de agosto a novembro de 2024 e atualizado em março de 2025.

### Coleta de dados

Os dados secundários foram obtidos pelo sítio eletrônico Sistema de Informação de Agravos de Notificação (SINAN), por meio do DATASUS da Secretaria de Vigilância em Saúde do Ministério da Saúde (https://datasus.saude.gov.br/informacoes-de-saude-tabnet/).

Definição da população Foram incluídas no estudo mulheres com diagnóstico de coinfecção TB/HIV, com a variável AIDS codificada como “sim” nas notificações de coinfecção TB/HIV registradas no SINAN e residentes em regiões pertencentes aos estados brasileiros no momento do diagnóstico. Foram excluídos os casos ignorados e/ou que se encontravam em branco.

### Variáveis do estudo

A variável dependente para nossa análise de séries temporais foi o número anual de casos de coinfecção TB/HIV em mulheres, estratificado por cada uma das cinco grandes regiões do Brasil (Norte, Nordeste, Sudeste, Sul e Centro-Oeste). No SINAN, a coinfecção TB/HIV é definida como o diagnóstico de TB em indivíduos com infecção concomitante por HIV.

Como variáveis independentes, consideramos o ano de notificação (para a análise de tendência temporal) e um conjunto de variáveis de caracterização, que dividimos em dois grupos. O primeiro grupo conteve as características sociodemográficas e comorbidades, como faixa etária (< 1 ano, 1 a 19, 20 a 39, 40 a 59, 60 anos ou mais), raça/cor (branco, preta, amarela, indígena), escolaridade (iletrado, até oito anos de estudo ou oito anos ou mais), transtorno por consumo de álcool (sim/não), transtorno por consumo de substância ilícita (sim/não), transtorno mental (sim/não) e transtorno por consumo de tabaco (sim/não). Já o segundo grupo conteve as seguintes características clínicas: tipo de entrada é caracterizado pela forma de ingresso do caso no sistema de notificação (caso novo, recidiva, transferência, pós-óbito); forma clínica inclui classificação da localização anatômica da TB (pulmonar, extrapulmonar, ou pulmonar e extrapulmonar); confirmação laboratorial refere-se à presença (com confirmação) ou ausência (sem confirmação) de resultado laboratorial que confirme o diagnóstico; tratamento antirretroviral (TARV) inclui o registro do uso de terapia antirretroviral para pessoas que vivem com HIV (sim/não); situação de encerramento engloba o desfecho final do caso de TB no sistema (cura, abandono, óbito por TB, óbito por outras causas, transferência, tuberculose drogarresistente (TB-DR) ou outro desfecho).

### Análise dos dados

Realizaram-se as análises descritivas das frequências absolutas e relativas referentes às características sociodemográficas, comorbidades e perfil clínico, utilizando o software estatístico Statistical Package for the Social Sciences, versão 20, IBM.

Para analisar a tendência temporal da coinfecção TB/HIV em mulheres nas diferentes regiões do Brasil, usamos o método de regressão de Prais-Winsten^7^. Este modelo é amplamente empregado em análises de séries temporais para corrigir a autocorrelação dos resíduos, que é uma característica comum em dados epidemiológicos sequenciais, garantindo, assim, a validade das estimativas^7^. No modelo, o número anual de casos de coinfecção TB/HIV em mulheres em cada região foi a variável dependente, e o ano de notificação, a variável independente.

A tendência temporal foi expressa como Variação Percentual Anual (VPA), com Intervalos de Confiança de 95% (IC95%). A tendência foi interpretada com base no nível de significância de 5% (p < 0,05) e no IC95% da VPA. Tendência estacionária foi considerada quando o IC95% da VPA contém o zero e o valor de p é maior que 0,05, indicando que não houve variação significativa ao longo do tempo. Tendência crescente é classificada quando o IC95% da VPA não contém zero, o valor da VPA é positivo e p < 0,05. Tendência decrescente é classificada quando o IC95% da VPA não contém zero, o valor da VPA é negativo e p < 0,05. Todas as análises de séries temporais foram realizadas no software Stata, versão 14.

## RESULTADOS

Entre o período de 2012 a 2023, foram notificados 31.171 casos de coinfecção TB/HIV entre mulheres a nível nacional, sendo a região Sudeste a que apresentou o maior número de notificações, com 11.791 casos (37,8%), seguida da região Sul, com 7.958 casos (25,5%), Nordeste, com 6.788 casos (21,7%), Norte, com 3.340 casos (10,7%), e Centro-Oeste, com 1.294 casos (4,1%). Ao analisar a caracterização sociodemográfica e as comorbidades associadas às regiões de notificações, a faixa etária de 20 a 39 anos e escolaridade até oito anos de estudo foram as variáveis mais prevalentes nas cinco regiões do Brasil. A cor preta apresentou maior notificações nas regiões Norte, Nordeste, Sudeste e Centro-Oeste, e a cor branca, na região Sul. Quanto às comorbidades, na região Centro-Oeste, o transtorno por consumo de álcool foi mais prevalente (24,5%), seguido do transtorno por consumo de tabaco (29,8%). Na região Sul, o transtorno por consumo de substâncias ilícitas (36,7%) e por tabaco (39,5%) foio mais prevalente, conforme descrito na Tabela 1.

**Tabela 1:** Caracterização do perfil sociodemográfico e comorbidades associadas à coinfecção TB/HIV em mulheres de acordo com as regiões do Brasil, 2012-2023

Variáveis	Norte n(%)	Nordeste n(%)	Sudeste n(%)	Sul n(%)	Centro-Oeste n(%)
**Faixa etária (anos)** *					
<1 ano	7(0,2)	47(0,7)	31(0,3)	12(0,2)	4(0,3)
1-19	140(4,2)	235(3,5)	376(3,2)	156(2,0)	23(1,8)
20-39	1784(53,4)	3677(54,2)	5890(50,0)	4273(53,7)	634(49,0)
40-59	1257(37,6)	2550(37,6)	4905(41,6)	3163(39,7)	546(42,2)
>60	152(4,6)	279(4,1)	589(5,0)	354(4,4)	87(6,7)
**Escolaridade (anos de estudo)** *					
Iletrado	121(4,7)	615(13,6)	718(8,6)	403(6,3)	78(8,5)
<8	1507(58,0)	2918(64,5)	4622(55,1)	4538(71,5)	558(60,5)
>8	1069(41,2)	989(21,9)	3042(36,3)	1410(22,2)	286(31,0)
**Raça/cor** *					
Branca	240(7,4)	639(10,2)	3429(32,1)	4650(59,8)	270(21,8)
Preta	2947(91,2)	5559(89,0)	7165(67,0)	3089(39,7)	935(75,6)
Amarela	20(0,6)	36(0,6)	74(0,7)	34(0,4)	7(0,6)
Indígena	23(0,7)	11(0,2)	26(0,2)	8(0,1)	25(2,0)
**Transtorno por consumo de álcool** *					
Sim	504(16,0)	1412(23,7)	2026(18,7)	1625(21,2)	276(24,5)
Não	2641(84,0)	4546(76,3)	8807(81,3)	6042(78,8)	849(75,5)
**Transtorno por consumo de substância ilícita** *					
Sim	441(17,6)	1236(26,7)	2607(27,1)	2198(36,7)	228(25,1)
Não	2067(82,4)	3398(73,3)	7030(72,9)	3788(63,3)	679(74,9)
**Transtorno por consumo de tabaco** *					
Sim	413(16,4)	1092(23,9)	2086(21,7)	2352(39,5)	270(29,8)
Não	2099(83,6)	3476(76,1)	7506(78,3)	3595(60,5)	635(70,2)

* *Variáveis ignoradas e em branco foram excluídas da análise*.

Na Tabela 2, é observado o perfil clínico da coinfecção TB/HIV. Assim, caso novo, forma clínica pulmonar e com TARV foram mais prevalentes nas cinco regiões do Brasil. Quanto à confirmação laboratorial da TB, apenas as regiões Sudeste e Sul apresentaram confirmação por laboratório. Referente ao encerramento dos casos, a cura foi evidenciada em maior proporção em todas as cinco regiões, no entanto o abandono e TB-DR foram mais prevalentes na região Sul (27,9% e 2,0%, respectivamente), óbito por TB, na região Sudeste (4,9%), e óbitos por outras causas, na região Centro-Oeste (21,1%).

**Tabela 2:** Perfil clínico da coinfecção TB/HIV em mulheres de acordo com as regiões do Brasil, 2012-2023

Variáveis	Norte n(%)	Nordeste n(%)	Sudeste n(%)	Sul n(%)	Centro-Oeste n(%)
**Tipo de entrada** *					
Caso novo	2478(74,3)	4346(64,5)	7972(68,1)	4809(60,7)	951(74,3)
Recidiva	728(21,8)	1936(28,7)	3260(27,8)	2683(33,8)	262(20,5)
Transferência	114(3,4)	391(5,8)	319(2,7)	386(4,9)	58(4,5)
Pós-óbito	15(0,4)	63(0,9)	157(1,3)	50(0,6)	9(0,7)
**Forma clínica** *					
Pulmonar	2328(69,7)	4932(72,7)	8327(70,6)	5444(68,4)	847(65,5)
Extrapulmonar	569(17,0)	1318(19,4)	2262(19,2)	1601(20,1)	311(24,0)
Pulmonar + extrapulmonar	443(13,3)	538(7,9)	1201(10,2)	913(11,5)	136(10,5)
**Confirmação laboratorial** *					
Com confirmação	1545(46,3)	3004(44,3)	6221(52,8)	4460(56,0)	537(41,5)
Sem confirmação	1795(53,7)	3784(55,7)	5570(47,2)	3498(44,0)	757(58,5)
**Tratamento antirretroviral** *					
Sim	1582(80,1)	2592(72,5)	4471(74,5)	3431(66,1)	667(81,5)
Não	392(19,9)	984(27,5)	1529(25,5)	1759(33,9)	151(18,5)
**Situação de encerramento** *					
Cura	1483(46,5)	2293(35,2)	5249(46,0)	3077(39,3)	440(35,9)
Abandono	677(21,2)	1673(25,7)	2570(22,5)	2189(27,9)	270(22,0)
Óbito por tuberculose	86(2,7)	303(4,7)	555(4,9)	99(1,3)	43(3,5)
Óbito por outras causas	578(18,1)	980(15,0)	2091(18,3)	1352(17,2)	258(21,1)
TB-DR**	47(1,5)	58(0,9)	164(1,4)	158(2,0)	12(1,0)
Outro desfecho	321(10,1)	1207(18,5)	779(6,8)	964(12,3)	202(16,5)

* *Variáveis ignoradas e em branco foram excluídas da análise; TB-DR - tuberculose drogarresistente*.

A técnica de decomposição de séries temporais (Figura 1) mostrou um aumento na tendência temporal da coinfecção TB/ HIV estacionária para todas as regiões do Brasil. Esses achados corroboram os dados apresentados na Tabela 3 referentes aos resultados da análise de Prais-Winsten. Ademais, por meio da autorregressão Prais-Winsten, no período de 2012 a 2023, foi possível observar que a coinfecção TB/ HIV permaneceu com uma tendência estacionário, observando uma maior taxa de coinfecção na região Sul, seguida da Norte, conforme descrito na Tabela 3.

**Figura 1:** Decomposição de séries temporais da coinfecção TB/HIV no Brasil: tendência crescente estacionária em todas as regiões, 2012-2023

## DISCUSSÃO

Este estudo teve como propósito identificar o perfil clínico operacional e classificar a tendência temporal entre as mulheres com coinfecção TB/HIV nas regiões do Brasil, de modo a subsidiar estratégias de prevenção e controle direcionadas à atuação de órgãos formuladores de políticas públicas.

A análise dos 31.171 casos notificados de coinfecção TB/HIV entre mulheres no Brasil evidenciou uma expressiva concentração na região Sudeste, responsável por 37,8% dos registros, seguida das regiões Sul (25,5%) e Nordeste (21,7%). Todavia, também esse índice pode ser reflexo da maior densidade populacional da região e de uma estrutura de notificação e vigilância em saúde mais consolidada.

Ao analisar a distribuição desigual de casos encontrada, tornase possível evidenciar não apenas as disparidades regionais na detecção da coinfecção TB/HIV, mas o caráter estrutural da vulnerabilidade social enfrentada por essas mulheres brasileiras^(8)^.

Além do mais, o cenário da coinfecção TB/HIV está intrinsecamente ligado a condições de pobreza, baixa escolaridade, ausência de rede de apoio e exclusão do sistema de saúde, o que potencializa a morbimortalidade entre as mulheres, sobretudo as jovens(9,10).

Nesse contexto, é importante considerar que a maior proporção de notificações de coinfecção TB/HIV entre mulheres pode ser explicada por aspectos do seu processo sócio-histórico, marcado por uma maior valorização do autocuidado e uma busca mais frequente pelos serviços de saúde em comparação aos homens. Esse comportamento favorece o diagnóstico precoce e o início oportuno do tratamento. Estudos realizados na África do Sul e em Uganda corroboram essa diferença de desfecho entre os sexos, indicando que os homens apresentam maior probabilidade de evoluir para óbito em decorrência da TB quando comparados às mulheres(11,12).

Desse modo, a identificação do perfil clínico-operacional dessas mulheres se torna, portanto, essencial para o redirecionamento das políticas públicas e formulação de estratégias mais equitativas e sensíveis às especificidades locais e de gênero.

No que diz respeito à faixa etária dos participantes do estudo, que incluiu a região Sudeste, observou-se uma concentração entre 20 e 39 anos. Corroborando esse achado, estudo realizado no estado do Rio de Janeiro, Brasil, entre os anos de 2000 e 2016, também identificou uma maior proporção de casos nessa mesma faixa etária^13^. Já a nível internacional, estudo na África Subsaariana, por sua vez, registrou uma maior prevalência na faixa etária dos 30 a 49 anos em mulheres com coinfecção TB/HIV^(14)^.

Essa concentração em mulheres mais jovens pode estar relacionada ao período de maior atividade hormonal, que está associado ao aumento do desejo sexual e à maior frequência de relações sexuais. Tais características ampliam a exposição a fatores de risco, como múltiplos parceiros, dificuldade na negociação do uso de preservativos, violência sexual e uso de substâncias psicoativas, condições que favorecem a infecção pelo HIV^(15)^.

No que diz respeito à escolaridade, observou-se que a maior proporção de notificações ocorreu entre mulheres com até oito anos de estudo. Esse grupo tende a ter menor acesso a informações essenciais sobre a prevenção da TB e do HIV, o que favorece comportamentos de risco, como a prática de sexo desprotegido e a dificuldade em reconhecer sintomas precoces da infecção^(6)^. Ademais, níveis mais baixos de escolaridade estão associados a uma maior exposição a fatores de vulnerabilidade, como as relações sexuais sem proteção, o que eleva o risco não apenas de gravidez indesejada, mas também de infecções sexualmente transmissíveis, incluindo o HIV^(16)^.

Na Índia, evidenciou-se que mulheres pertencentes a comunidades com maior nível de riqueza, escolaridade e emprego apresentavam maior conhecimento sobre o vírus do HIV, o que esteve associado a menores taxas de infecção^17^. Esses achados, que espelham realidades de desigualdade socioeconômica e barreiras culturais presentes em países de grande dimensão e diversidade como o Brasil, reforçam que tais barreiras limitam significativamente o acesso aos serviços de saúde, contribuindo para diagnósticos tardios e menor adesão ao tratamento. Além disso, a baixa escolaridade está frequentemente associada a condições de vida precárias, que favorecem a disseminação da TB, bem como ao estigma social, que dificulta a busca por cuidados adequados^(17)^.

No contexto brasileiro, observam-se padrões semelhantes, em que as maiores taxas de coinfecção frequentemente recaem sobre populações mais vulneráveis socioeconomicamente, incluindo mulheres negras, apesar de o contingente populacional branco ser maior, o que exige a compreensão das diferenças contextuais para o enfrentamento da doença^18^. Essas vulnerabilidades se refletem nas regiões com maiores notificações e indicam uma tendência temporal ligada à exclusão social. O estigma e as barreiras estruturais dificultam a busca por cuidados, e ampliam a morbimortalidade. Assim, estratégias de enfrentamento devem considerar essas desigualdades para qualificar a resposta à coinfecção^19^.

No Brasil, a população de raça/cor não branca foi menos acometida pela coinfecção TB/HIV, padrão também observado em países como Estados Unidos e Porto Rico^20^. Essa maior suscetibilidade está associada a contextos de vulnerabilidade socioeconômica, nos quais populações negras enfrentam barreiras estruturais, como o acesso limitado a serviços de saúde e educação, além da persistente discriminação racial^21^. Ademais, estudos indicam que, no Brasil, a probabilidade de vivência deviolência sexual na vida adulta é significativamente maior entre mulheres negras em comparação a outros grupos. Essa situação não apenas as expõe a um maior risco de infecção pelo HIV, mas também pode criar barreiras significativas ao acesso e à adesão ao tratamento e prevenção da coinfecção TB/HIV, influenciando negativamente os desfechos de saúde^(22)^.

**Tabela 3:** Tendência temporal da coinfecção TB/HIV em mulheres de acordo com as regiões do Brasil, 2012-2023

Região	Notificações	Taxa	VPA (IC95%)	Valor de *p*	Tendência
Norte	3296	37,92	26,38 (-1,20 - 80,27)	0,172	Estacionária
Nordeste	6611	23,41	1,03 (-1,55 - 3,68)	0,398	Estacionária
Sudeste	11494	26,13	-0,09 (-0,22 - 0,02)	0,096	Estacionária
Sul	7899	51,45	-0,07 (-0,20 - 0,05)	0,209	Estacionária
Centro-Oeste	1267	15,29	5,83 (-2,64 - 15,04)	0,161	Estacionária

VPA - Variação Percentual Anual; IC95% - Intervalo de Confiança de 95%.

Apesar das diretrizes nacionais e internacionais para o controle da TB, a persistência de novos casos evidencia limitações significativas nos sistemas de vigilância e monitoramento, bem como nos determinantes sociais que dificultam o acesso ao diagnóstico e ao tratamento oportuno^23^. Esses aspectos foram evidenciados pelos resultados do presente estudo, que apontam não apenas para a continuidade da cadeia de transmissão, mas também para a complexidade envolvida na erradicação da doença em contextos de vulnerabilidade.

A forma extrapulmonar da TB apresenta maior prevalência entre pessoas vivendo com HIV, uma vez que a imunossupressão favorece a disseminação do bacilo para além dos pulmões^24^. Além disso, 27% das mulheres com a infecção TB/HIV apresentaram essa forma clínica, o que reforça a necessidade de atenção especializada e estratégias diagnósticas sensíveis para identificação precoce desses casos. Tal cenário exige não apenas o fortalecimento das ações de vigilância epidemiológica, mas também a ampliação da cobertura dos serviços de saúde e da qualificação das equipes para o manejo clínico-operacional da coinfecção, sobretudo em mulheres em situação de vulnerabilidade social.

Apesar dos notáveis avanços no tratamento combinado da coinfecção TB/HIV, que incluem regimes eficazes para a TB e a ampla disponibilidade da TARV, essenciais para o alcance da cura, a interrupção terapêutica ainda é um desafio crítico. Mesmo com progressos observados em regiões como o Centro-Oeste, a descontinuidade está frequentemente ligada a fatores pessoais, como o transtorno relacionado ao consumo de álcool, tabaco, uso de drogas ilícitas, e a falhas estruturais nos serviços de saúde, como dificuldade de acesso e baixa cobertura do tratamento. Esse cenário favorece o surgimento de formas resistentes à doença. Além disso, o HIV é um preditor importante de interrupção do tratamento e mortalidade. Comorbidades como diabetes, transtornos mentais e uso de substâncias aumentam a vulnerabilidade das mulheres para realização do ato sexual sem o preservativo, bem como para a coinfecção TB/HIV, exigindo uma abordagem mais integrada e humanizada no cuidado^(25)^.

As disparidades regionais têm um impacto direto na incidência e nos desfechos da coinfecção TB/HIV no Brasil, sendo mais evidentes nas regiões Norte e Nordeste, historicamente menos desenvolvidas. Devido à dimensão continental do país, esses desequilíbrios são agravados por fatores como o acesso limitado a serviços de saúde, a carência de profissionais qualificados, a distribuição inadequada de medicamentos e insumos, e as dificuldades impostas por barreiras geográficas e socioeconômicas, contribuindo para a persistência das iniquidades^(26)^.

A escassez de estudos ecológicos sobre a distribuição espacial da coinfecção TB/HIV, especificamente entre mulheres, limita a compreensão aprofundada do problema em nível nacional. Contudo, os dados obtidos neste estudo demonstram claramente as desigualdades regionais no Brasil, reforçando a importância de direcionar mais pesquisas para essa temática. É fundamental destacar que, apesar da existência de programas robustos para o cuidado do HIV e da TB, que oferecem tratamento direcionado e acompanhamento, ainda persistem desafios na superação dessas iniquidades regionais e na garantia do acesso equitativo ao cuidado.

É importante considerar que, para os contingentes mais jovens e mais idosos das faixas etárias estudadas, as particularidades do cuidado, incluindo a necessidade de um responsável, são cruciais. Assim, é fundamental considerar aspectos subjetivos, geográficos e socioeconômicos para ampliar a adesão ao tratamento e fortalecer as políticas públicas.

### Limitações do estudo

As limitações do presente estudo incluem o fato de ser uma análise retrospectiva baseada em dados secundários do SINAN. Embora o SINAN seja uma fonte oficial valiosa, a qualidade e a completude das informações dependem do preenchimento no momento da notificação, o que pode levar a variáveis incompletas ou ausentes. Essa característica intrínseca da base de dados, somada à possibilidade de subnotificação, pode resultar em uma subestimação da real magnitude do problema e, consequentemente, afetar a generalização dos achados. Adicionalmente, deve-se considerar a inerente limitação de estudos ecológicos na inferência individual, o que exige cautela na interpretação para evitar a falácia ecológica. Por fim, a demora na disponibilização de dados mais recentes no sistema pode ter impactado a temporalidade das informações analisadas.

### Contribuições para a área da saúde, enfermagem ou políticas públicas

Este estudo contribui para a saúde pública ao evidenciar os determinantes sociais, clínicos e regionais que influenciam a coinfecção TB/HIV entre as mulheres brasileiras, orientando políticas mais equitativas e regionalizadas. Para a enfermagem, destaca-se a importância do seu papel no diagnóstico precoce, adesão ao tratamento e acompanhamento contínuo, sobretudo em contextos de vulnerabilidade, uma vez que o enfermeiro tem papel primordial no gerenciamento dos serviços de saúde e no plano de cuidado a essa população.

## CONCLUSÕES

A coinfecção TB/HIV entre mulheres no Brasil apresenta disparidades regionais e está fortemente associada a fatores sociodemográficos como baixa escolaridade, faixa etária entre 20 e 39 anos, e raça/cor preta, especialmente nas regiões Norte, Nordeste, Sudeste e Centro-Oeste. A região Sudeste concentrou o maior número de notificações, mas foi na região Sul que se observaram as maiores taxas de interrupção e resistência medicamentosa, bem como maior prevalência de transtornos por uso de substâncias.

O perfil clínico predominante foi de casos novos, forma pulmonar e em uso de TARV. A tendência temporal da coinfecção permaneceu estacionária entre 2012 e 2023, com destaque para a elevação da taxa nas regiões Sul e Norte. Portanto, os achados desta pesquisa fornecem contribuições valiosas para o desenvolvimento de políticas públicas direcionadas às mulheres, considerando as particularidades da coinfecção TB/HIV de cada região brasileira.

## Data Availability

Os dados da pesquisa estão disponíveis no corpo do artigo.
